# Proteomic identification of secreted proteins of *Propionibacterium acnes*

**DOI:** 10.1186/1471-2180-10-230

**Published:** 2010-08-27

**Authors:** Carsten Holland, Tim N Mak, Ursula Zimny-Arndt, Monika Schmid, Thomas F Meyer, Peter R Jungblut, Holger Brüggemann

**Affiliations:** 1 Department of Molecular Biology, Max Planck Institute for Infection Biology, Berlin, Germany; 2 Proteomics Core Facility, Max Planck Institute for Infection Biology, Berlin, Germany

## Abstract

**Background:**

The anaerobic Gram-positive bacterium *Propionibacterium acnes *is a human skin commensal that resides preferentially within sebaceous follicles; however, it also exhibits many traits of an opportunistic pathogen, playing roles in a variety of inflammatory diseases such as *acne vulgaris*. To date, the underlying disease-causing mechanisms remain ill-defined and knowledge of *P. acnes *virulence factors remains scarce. Here, we identified proteins secreted during anaerobic cultivation of a range of skin and clinical *P. acnes *isolates, spanning the four known phylogenetic groups.

**Results:**

Culture supernatant proteins of *P. acnes *were separated by two-dimensional electrophoresis (2-DE) and all Coomassie-stained spots were subsequently identified by MALDI mass spectrometry (MALDI-MS). A set of 20 proteins was secreted in the mid-exponential growth phase by the majority of strains tested. Functional annotation revealed that many of these common proteins possess degrading activities, including glycoside hydrolases with similarities to endoglycoceramidase, β-N-acetylglucosaminidase and muramidase; esterases such as lysophospholipase and triacylglycerol lipase; and several proteases. Other secreted factors included Christie-Atkins-Munch-Petersen (CAMP) factors, glyceraldehyde 3-phosphate dehydrogenase (GAPDH), and several hypothetical proteins, a few of which are unique to *P. acnes*. Strain-specific differences were apparent, mostly in the secretion of putative adhesins, whose genes exhibit variable phase variation-like sequence signatures.

**Conclusions:**

Our proteomic investigations have revealed that the *P. acnes *secretome harbors several proteins likely to play a role in host-tissue degradation and inflammation. Despite a large overlap between the secretomes of all four *P. acnes *phylotypes, distinct differences between predicted host-tissue interacting proteins were identified, providing potential insight into the differential virulence properties of *P. acnes *isolates. Thus, our data presents a rich resource for guiding much-needed investigations on *P. acnes *virulence factors and host interacting properties.

## Background

The Gram-positive skin commensal *Propionibacterium acnes *is ubiquitously present on human skin. It has been speculated that this bacterium contributes to healthy skin by deterring the colonization of severe pathogens [1,2]; however, it is most well known for its role in skin disorders such as *acne vulgaris *[3,4]. Acne, a multifactorial disorder related to the formation of comedones, hormonal stimulation, bacterial colonization and the host inflammatory response, is an extremely common condition affecting approximately 80% of adolescents. Despite intense research effort, the precise role of *P. acnes *in acne formation is still unclear [5-7].

In addition to acne, *P. acnes *has been frequently associated with a variety of inflammatory diseases, including prosthetic joint infections, shunt-associated central nervous system infections, endocarditis, sarcoidosis, endophthalmitis, osteomyelitis, allergic alveolitis, pulmonary angitis, acne inversa (alias hidradenitis suppurativa), and the SAPHO (synovitis, acne, pustulosis, hyperostosis, osteitis) syndrome [8-10]. This bacterium is also a common isolate of prostatic glands from patients with prostate inflammation [11,12]. Interestingly, the role of *P. acnes *in the development of prostate cancer through an inflammatory mechanism is currently a subject of much speculation [12-14].

The prevalence of *P. acnes *in the above-mentioned conditions suggests that this bacterium is an etiological agent of infection and that it possesses an elevated pathogenic potential. *P. acnes *has been shown to exhibit haemolytic and cytotoxic activities [15,16] as well as extensive immunostimulatory activity and complement activation [6,17-20]. *P. acnes *isolates differ in their virulence properties, such as in their ability to trigger production of proinflammatory cytokines/chemokines in infected keratinocytes [21,22]. The genetic basis for this has not yet been studied in detail. To date four phylogenetic groups of *P. acnes *have been described, designated types IA, IB, II and III, based on sequence differences in two genes, namely *recA *and *tly *[23,24].

Despite the apparent role of *P. acnes *in disease formation, information on putative pathogenic traits and antigenic substances of this bacterium is scarce. The complete genome sequence of a cutaneous type IB isolate of *P. acnes *(strain KPA171202) provided insights into the pathogenic potential of *P. acnes*, revealing numerous gene products with putative host tissue-degrading activities as well as predicted cell wall-associated and secreted proteins, the presence or activity of which might be involved in triggering host tissue inflammation [25]. Some of these proteins are differentially expressed among *P. acnes *isolates and were shown to be immunoreactive [26].

To shed light on the biological relevance of predicted genes from the genome sequence, we used a combination of two-dimensional electrophoresis (2-DE) and matrix-assisted-laser-desorption/ionization mass spectrometry (MALDI-MS) to identify proteins secreted by *P. acnes*. Isolates representing all four phylotypes were investigated. Several hydrolases and putative virulence factors were secreted by all strains tested. These factors are potential host-interacting factors, likely important in inflammatory responses to *P. acnes*, as observed in *acne vulgaris*. Thus, our data provide a basis to guide further in-depth studies on individual factors.

## Results and Discussion

### Choice of *P. acnes *strains

We selected five strains of *P. acnes *for analysis of their secreted proteins. These strains, representing all known *P. acnes *phylotypes, i.e. types IA, IB, II and III, were isolated from a range of tissue sites: a type II skin acne isolate (strain 329); a type III strain isolated from a post-operative prosthetic joint infection (strain 487); a type IA strain isolated from a pleuropulmonary infection (strain 266); and two type IB strains: a skin isolate for which the genome sequence is available (strain KPA171202; KPA); and an isolate from a cancerous prostate (strain P6).

### 2-DE-MALDI-MS analysis of *P. acnes *culture supernatants

**Figure 1:**
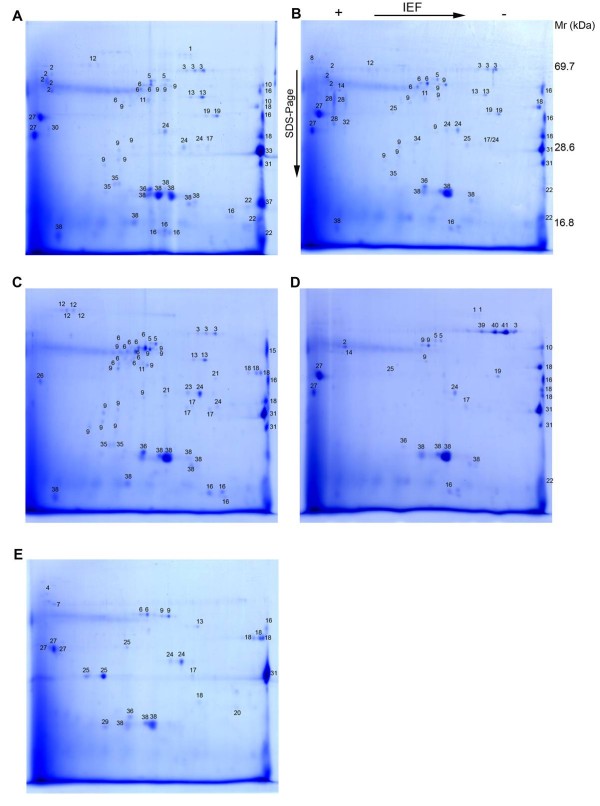
**2-DE of *P. acnes *culture supernatants**. Bacteria were grown in BHI medium to an OD_600 _of 0.6. Supernatants were harvested and precipitated. Protein samples (200 μg) from each strain were separated on 2-DE gels and visualized by staining with Coomassie Brilliant Blue G-250. The following strains were used: (a) KPA171202 (type IB); (b) P6 (type IB); (c) 266 (type IA); (d) 329 (type II); (e) 487 (type III). Information about the identified protein spots is provided in additional file 2.

To identify proteins secreted by *P. acnes *using proteome analysis, we cultured each strain under anaerobic conditions in brain heart infusion (BHI) broth, previously used for secretome analyses [27]. Growth curves were generated (data not shown) and culture supernatants were harvested in the mid-exponential phase. Precipitated proteins from supernatants were separated by 2-DE and Coomassie stained, generating reproducible secretomes of all five strains tested (Fig. 1A-E and additional file 1). All visible protein spots were analyzed by MALDI-MS and searched against the NCBI non-redundant database, which included (at the time of analysis) the genome sequence of the type IB strain KPA and the partial genome sequence of the type IA strain SK-137.

The identified proteins for each strain, with molecular weights, isoelectric points, Mascot scores and sequence coverage are listed in additional file 2. In total, 64, 63, 54, 30, and 28 protein spots for *P. acnes *strains 266, KPA, P6, 329 and 487, respectively, were unambiguously identified and assigned to database entries. Several proteins occurred in spot series, representing different protein species of the same protein. Post-translational modifications are a likely explanation, resulting in altered molecular masses and/or isoelectric points [28]. A few MS spectra originating from secreted proteins of strain 329 could not be assigned to any database entry (Fig. 1D, spots 39-41), indicating that these proteins are strain-specific. The inability to identify these proteins also reflects the absence of genome sequence data from type II and type III strains; only genome sequences from type I strains are currently available.

### Twenty commonly secreted proteins of *P. acnes*

**Figure 2:**
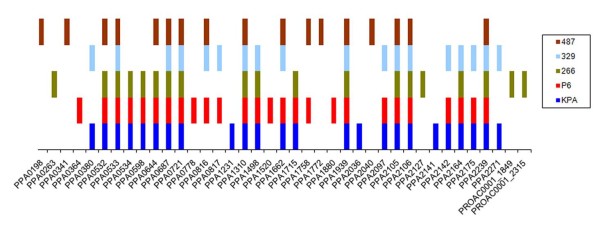
**Distribution of secreted proteins in five *P. acnes *strains**. The identified proteins in each strain were assigned to the gene nomenclature of the KPA genome (PPA numbers) and of the partial genome of SK137 (PROAC numbers).

The identified proteins secreted by the five strains tested were assigned to the reference KPA genome (Fig. 2, additional file 2). A set of 20 proteins was secreted by at least three of the five strains, including eight proteins secreted by all strains (Table 1). All 20 proteins were secreted by the P6 strain, whereas 19 (95%), 15 (75%), 15 (75%) and 12 (60%) of these proteins were secreted by the KPA, 266, 329 and 487 strains, respectively. We cannot exclude, however, that proteins secreted at lower levels were missed by our approach, as the amount of secretion varied between the strains and the sensitivity of the Coomassie stain is limited to the 100 ng range.

**Table 1:** Twenty proteins constitute the common secretome of *P. acnes*.

Gene ID	Updated annotation	Protein family/domains/origin of closest ortholog	Secretion signal^a^	Phylo-type	Semi-quantification^b^/comments
PPA0532	conserved hypothetical protein	*Corynebacterium*	SP, TAT	IA, IB, III	++

PPA0533	conserved hypothetical protein	*Corynebacterium*	SP	IA, IB, II, III	+

PPA0534	conserved hypothetical protein	*Corynebacterium*	SP	IA, IB	+

PPA0598	putative protease	peptidase S8/S53 superfamily; *Arthrobacter*	SP, (TAT)	IA, IB	+

PPA0644	putative endo-glycoceramidase	cellulase, glycoside hydrolase family 5; *Corynebacterium*	(SP), TAT	IA, IB, III	++/42% identity to PPA2106

PPA0687	CAMP2	CAMP factor superfamily; *Mobiluncus curtisii, Streptococcus*	SP	IA, IB, II, III	+++

PPA0721	putative invasion-associated protein; NlpC/P60 family	NlpC/P60 family; *Kribbella flavida, Streptomyces*	SP, TAT	IA, IB, II, III	+++/NlpC/P60 is found in cell wall hydrolases

PPA0816	glyceraldehyde 3-phosphate dehydrogenase	GAPDH	no	IB, II, III	+

PPA1310	putative protease	PDZ superfamily; *Kribbella flavida, Streptomyces*	Internal SP (wrong N-terminus)	IA, IB, II, III	+/PDZ is a signaling domain

PPA1498	putative phosphoesterase	metallo-dependent phosphatase superfamily; *Rhodococcus*	TAT	IA, IB, II	+

PPA1662	putative lysozyme	CH-type (chalaropsis-type) lysozyme, glycoside hydrolase family 25; *Streptomyces*	SP, TAT	IB, II, III	+++

PPA1715	hypothetical protein, specific to *P. acnes*		SP	IA, IB	+/17 PT repeats

PPA1939	hypothetical protein, specific to *P. acnes*		SP	IA, IB, II, III	+++

PPA2097	putative 5'-nucleotidase, metallo-phosphoesterase	UshA (5'-nucleotidase/2',3'-cyclic phosphodiesterase and related esterases); *Jonesia denitrificans*	SP, TAT	IB, II	+

PPA2105	triacylglycerol lipase	lipase class 2, esterase/lipase superfamily; *Rhodococcus*	SP	IA, IB, II, III	++

PPA2106	putative endoglycoceramidase	cellulase, glycoside hydrolase family 5; *Nocardioides*	SP, (TAT)	IA, IB, II, III	+/42% identity to PPA0644

PPA2142	putative lysophospholipase	alpha/beta-hydrolase superfamily, PldB; *Corynebacterium*	SP, TAT	IB, II	+

PPA2164	putative beta-N-acetyl-glucosaminidase	glycoside hydrolase family 3; *Arthrobacter*	SP	IA, IB, II	++

PPA2175	hypothetical protein, with SH3 and RlpA domains, specific to *P. acnes*	SH3 (type 3) domain; peptidoglycan-binding domain; C-terminus: DPBB_1 (RlpA-like double-psi beta-barrel)	SP, TAT	IB, II	+++/SH3: Src homology 3 domain

PPA2239	putative peptidase/glycosyl hydrolase	DUF348 superfamily; G5 domain; C-terminus: DPBB_1 (RlpA-like double-psi beta-barrel); *Janibacter*	SP	IA, IB, II, III	++/G5: a potential N-acetylglucosamine recognition domain

At least three of the five *P. acnes *strains secrete the listed proteins; see additional file 2 for a complete list of identified proteins. Re-annotation was performed for each gene. ^a ^SP, signal peptide; TAT, twin-arginine motif; ^b ^Semi-quantification based on Coomassie stained gels

All 20 proteins except one, glyceraldehyde 3-phosphate dehydrogenase (GAPDH), carried a secretion signal in its N-terminus. *P. acnes *has a general secretion (Sec)/signal recognition particle (SRP) and a twin-arginine translocation (TAT) secretion system. Other secretion systems have not been reported for *P. acnes*, and rescanning of the genome sequence gave no indication that additional ones exist.

### Hydrolytic enzymes are secreted by *P. acnes*

To gain insight into the biological functions of the 20 commonly secreted proteins, re-annotation was performed based on similarity searches against protein sequence and protein-domain/-family databases (Table 1). Many of the secreted proteins were found to have predicted hydrolytic activities: two genes (PPA0644 and PPA2106) are predicted endo-glycoceramidases, sharing 42% identity on the protein level. Although their substrate specificities are unknown, PPA0644 and PPA2106 share 27% and 30% protein identity, respectively, with the characterized and structurally analyzed endo-glycoceramidase II from *Rhodococcus *sp., which hydrolyzes glycosidic linkages between the oligosaccharide and ceramide moieties of gangliosides [29]. Another secreted protein, PPA2164, a glycoside hydrolase family 3 protein, shares 31% identity on the protein level with NagZ (formerly YbbD) of *B. subtilis*. NagZ is a β-N-acetylglucosaminidase involved in the peptidoglycan recycling pathway; it cleaves the terminal non-reducing N-acetylglucosamine of muropeptides [30]. *P. acnes *also secreted a putative lysozyme (PPA1662) which is 47% identical on the protein level to the muramidase from *Streptomyces coelicolor*. This muramidase not only cleaves the β-1,4-glycosidic bond between N-acetylmuramic acid and N-acetylglucosamine units, but also exhibits β-1,4-N,6-O-diacetylmuramidase activity, enabling this enzyme to degrade *Staphylococcus aureus *cell walls [31]. Whether PPA1662 is an autolytic lysozyme involved in cell wall turnover has still to be elucidated. However, the peptidoglycan of *P. acnes *contains non-N-acetylated glucosamine residues and is therefore resistant to lysozyme [32]. We speculate that PPA1662 has a different substrate specificity, acting on non-N-acetylated peptidoglycan, or, alternatively, it acts as a defense system against competing bacteria on the skin.

Two strains, KPA and 329, secreted a hyalorunate lyase (PPA0380), confirming previous investigations on a *P. acnes *protein with hyalorunate lyase activity [33,34]. Preliminary functional characterization revealed that the enzyme exerted activity against chondroitin 4- and 6-sulphates but not against dermatan sulphate [33]. In accordance, the closest characterized homolog, the chondroitin lyase of *Arthrobacter aurescens *(37% protein identity to PPA0380) acts on chondroitin sulfate but not on dermatan sulfate [35]. Similar to other chondroitin lyases, it is capable of cleaving hyaluronan, a non-sulfated glycosaminoglycan and a major component of the extracellular matrix of connective tissues.

Consistent with the known lipolytic activity of *P. acnes *[36], we identified lipolytic enzymes in the secretory fraction, including the previously characterized triacylglycerol lipase, designated glycerol-ester hydrolase A (GehA; PPA2105). GehA is recognized as one of the virulence factors involved in the pathogenesis of acne [37-39], and is thought to be the main enzyme responsible for the hydrolysis of sebum triacylglycerides, resulting in the release of glycerol and free fatty acids. The released fatty acids are thought to be inflammatory; they favor ductal hypercornification and increase adhesion between *P. acnes *and cells of the hair follicle, promoting colonization of *P. acnes *and biofilm formation [37,40-42]. Furthermore, GehA itself is a strong chemotactic factor [43]. Other secreted esterases identified include a putative lysophospholipase (PPA2142) and a putative phosphoesterase (PPA1498) with unknown specificities. Proteases, another class of secreted hydrolases, were also detected, e.g. a peptidase S8/S53 family protein (PPA0598) among others; their substrate specificities remain to be elucidated.

### CAMP factors and other secreted proteins

A set of five highly similar *P. acnes *genes (PPA687, PPA1198, PPA1231, PPA1340, PPA2108) in the genome of *P. acnes *KPA encodes homologs to Christie-Atkins-Munch-Petersen (CAMP) factors, which are co-haemolytic proteins, found mainly in streptococcal species [25,44,45]. CAMP factors have been characterized as pathogenic determinants that exert lethal effects when administered to rabbits and mice [46]. In addition, streptococcal CAMP factors have been reported to act as pore-forming toxins [47]. In agreement with previous work [45], all *P. acnes *strains examined here were positive for the co-haemolytic CAMP reaction (data not shown). Our secretome data showed that all tested *P. acnes *strains secreted CAMP2 (PPA0687). In addition, the skin isolate KPA secreted CAMP4 (PPA1231). Secretion of the other three CAMPs was not observed in any strain using our approach. A previous study reported variable production of CAMP factors in different *P. acnes *isolates, as detected by western blotting experiments using different anti-CAMP sera [45]; the authors reported an abundance of CAMP1 in type IB and II strains. We did not find CAMP1 among the secreted proteins; a discrepancy that could be due to the detection limits of the different techniques used, i.e. our MS analysis detects the most prominently secreted factors, whereas immunoblotting is a more sensitive technique.

A key enzyme of glycolysis, GAPDH, was also secreted by three out of the five *P. acnes *strains tested. At first glance it is peculiar why a glycolysis enzyme should be secreted; however, a number of studies have identified GAPDH as an anchorless, multifunctional protein, displayed on the surface of several fungi and Gram-positive pathogens, which contributes to adhesion and virulence [48,49]. In *Streptococcus pyogenes*, this cell-associated and soluble protein is also known as streptococcal surface dehydrogenase (SDH) and as a plasmin receptor (Plr); its complement C5a-binding activity was shown to play a role in evasion of neutrophil recruitment to sites of infection [50]. Moreover, in *S. agalactiae*, GAPDH is an immunomodulatory factor, exhibiting B lymphocyte-stimulatory activity [51].

In addition to the above-mentioned proteins, several (conserved) hypothetical secreted proteins were detected. Three of these hypothetical proteins are encoded by a gene cluster (PPA0532-0534), with homologs only in *Corynebacterium *spp. Three additional secreted proteins (PPA1715, PPA1939, PPA2175) are unique to *P. acnes*; PPA1715 contains characteristic repeats of the dipeptide proline-threonine (PT), similar to other putative adhesins (discussed below), and PPA1939 was secreted most strongly by all tested strains. Future work will determine the function of this abundantly secreted protein.

### Strain-specific secretion of putative adhesions

**Figure 3:**
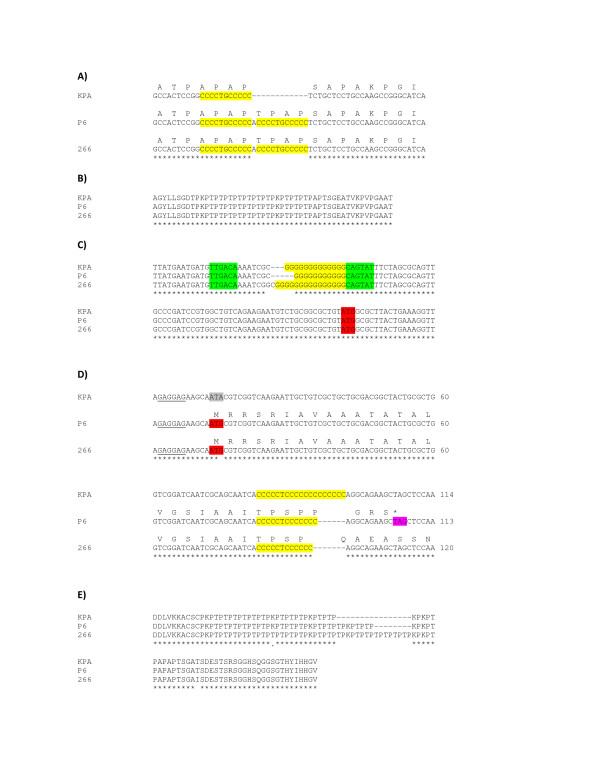
**Changes in repetitive sequences involved in strain-specific expression and secretion of putative adhesins of *P. acnes***. (a) Insertion of a 12 bp repeat in the 5'-end of PPA2141 in *P. acnes *strains P6 and 266 results in an altered N-terminus. PPA2141 is secreted only by strain KPA. (b) Proline-threonine (PT) repeats at the C-terminus of PPA1880; these repeats are conserved in the indicated *P. acnes *strains. (c) Changes in the number of guanine residues in the upstream region of PPA1880, resulting in altered sizes of the spacer region of the possible promoter (in green: putative -35 and -10 region of the promoter; in red: predicted start codon). An alternative consequence of these alterations is shown in Fig. S2. PPA1880 is only secreted by strain P6. (d) Sequence differences of PPA2127: mutated start codon in strain KPA and variation of the C tract in the 5' end of the gene. PPA2127 is only secreted by strain 266. (e) Different numbers of PT repeats at the C-terminus of PPA2127.

As expected, the secretomes of the type IB strains, KPA and P6, share a higher degree of similarity with each other than with the other three strains tested. Nevertheless, we identified a few prominent differences between KPA and P6: (i) KPA secreted both CAMP4 and CAMP2. By contrast, P6 exclusively secreted CAMP2; (ii) KPA was the only strain which secreted PPA2141, a protein unique to *P. acnes *and with no homology to proteins stored in any database. A likely explanation for the KPA-specific expression of the gene encoding PPA2141 is a duplication of a 12 bp repeat within the 5'-end of the gene in strains 266 and P6 (Fig. 3A). This duplication results in the insertion of four amino acids just after the predicted cleavage site of the signal peptide, which potentially alter secretion; (iii) likewise, PPA1880, which also has no existing homology to other proteins but contains characteristic PT repeats (Fig. 3B), was secreted exclusively by P6. Interestingly, PPA1880 possesses a phase variation-like signature - a stretch of guanine residues, located within the putative promoter region. Sequencing of the upstream region of PPA1880 revealed a variable number of guanine residues in the three strains (11 nt in P6, 13 nt in KPA and 15 nt in 266) (Fig. 3C). Changes in the number of guanine residues alter the length of the spacer region of the putative promoter. Thus, observed differences in spacer lengths - 18 nt in P6 (close to the consensus length), 20 nt in KPA and 23 nt in 266 - may explain why PPA1880 expression is P6-specific. Alternatively, if the guanine tract is assumed to be part of the N-terminus of PPA1880, frameshifts leading to truncated proteins would be introduced in KPA and 266, but not in P6 (additional file 3).

A number of major differences were detected between strains belonging to phylotypes IA and IB: In comparison to the two type IB strains (KPA and P6), strain 266, a type IA strain, exhibited (i) reduced lysozyme (PPA1662) secretion, and (ii) increased secretion of the lipase GehA; (iii) in addition, strain 266 exclusively secreted PPA2127. PPA2127 (also designated PA-25957) is a host cell-surface attachment protein with dermatan-sulphate-binding activity and has immunoreactive properties [26]. The corresponding gene is associated with a putative phase variation signature; variable expression in different *P. acnes *strains has been observed and attributed to mutated start codons or alterations in the length of the homopolymeric cytosine tract in the 5'-end of the gene [26]. Comparison of PPA2127 gene sequences from KPA, P6 and 266 revealed that the start codon was mutated in KPA. In strain P6 the length of the cytosine tract was altered, leading to a frameshift and the introduction of a premature stop codon (Fig. 3D). In addition, the number of PT repeats within the C-terminus of PPA2127 varied. These repeats were more numerous in strain 266 as compared to the two type IB strains (Fig. 3E).

Strain 329, a type II strain, secreted a few proteins (Fig. 1D, spots 39-41) which could not be assigned to any known protein. MALDI-MS identification and subsequent homology searches against the genomes of *P. acnes *and the whole NCBI database retrieved no significant matches, indicating that these proteins are unique to strain 329.

Strain 487, a type III strain, secreted fewer factors than any of the other strains. One protein, PPA1758, an outer membrane lipoprotein of the periplasmic binding proteins (PBPs) superfamily, was secreted solely by strain 487. PPA1758 exhibits a 38% protein identity to the membrane-associated glycylmethionine binding protein (GmpC) of *Staphylococcus aureus *[52], indicating a potential role as a dipeptide transporter for PPA1758.

### Secretome of *P. acnes *266 in stationary growth phase

**Figure 4:**
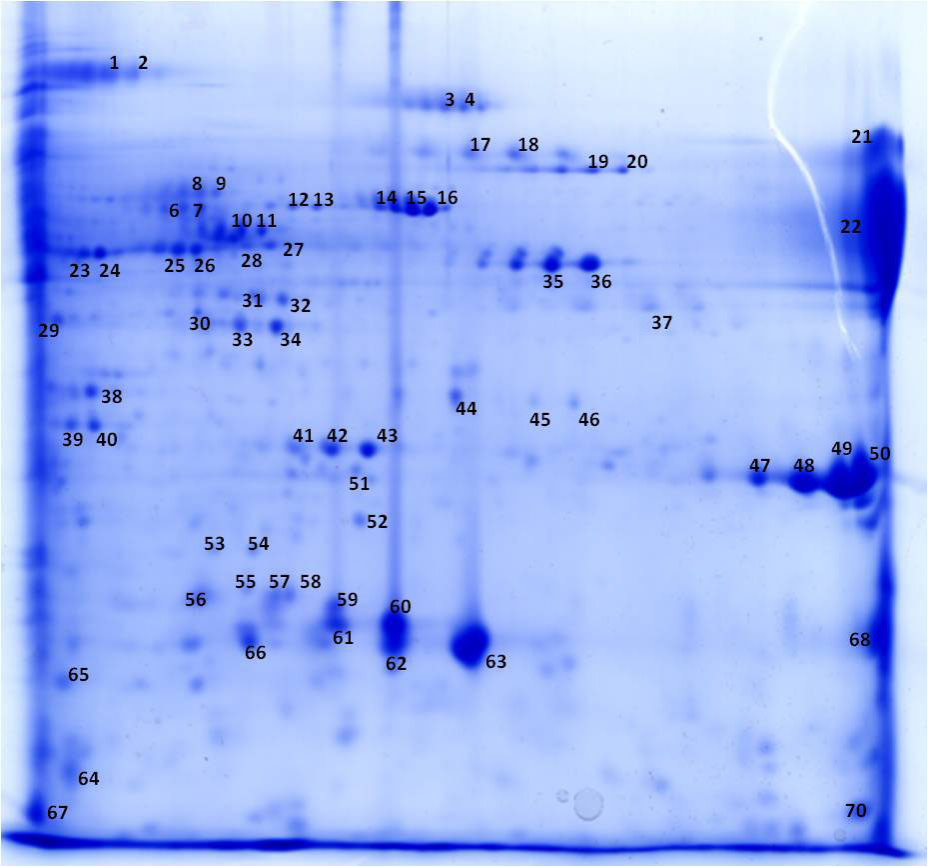
**Stationary phase secretome of *P. acnes *strain 266**. Strain 266 was grown in BHI medium for 72 h, culture supernatants were harvested and precipitated. Proteins were separated on a 2-DE gel and visualized by staining with Coomassie brilliant blue G-250. Information about the identified protein spots is provided in additional file 5.

To investigate growth phase-dependent secretion, *P. acnes *was grown to stationary phase. We selected strain 266 for this analysis as it was found to aggregate strongly upon reaching the stationary phase (additional file 4). 2-DE/MALDI-MS analysis of strain 266 culture supernatants revealed approximately half of the identified spots (33 out of 65) corresponded to proteins already identified as being secreted during the mid-exponential phase (Fig. 4 and additional file 5). The other spots corresponded to proteins mainly involved in key metabolic pathways and that are known to be primarily located in the bacterial cytoplasm. Thus, it is most likely that lysis of *P. acnes *occurred in the stationary phase, releasing the most abundant cytosolic proteins. Enzymes of key pathways such as glycolysis, pyruvate metabolism and the tricarboxylic acid cycle were identified, including phosphoglyceromutase, phosphoglycerate kinase, oxaloacetate decarboxylase, fumarate hydratase, and succinyl-CoA synthetase. In addition, we detected amino acid-converting proteins, i.e. serine hydroxymethyltransferase, tryptophanase and ornithine carbamoyltransferase. Other identified proteins included elongation factors, catalase, 10 kDa chaperonin as well as the fatty acid biosynthesis enzyme acyl-carrier-protein S-malonyltransferase. Only two proteins with a typical signal peptide, which were not detected in the exponential phase-secretome, were identified: PPA2152, an extracellular solute-binding protein, and PPA2210, another protein containing a long stretch of PT repeats. PPA2210, designated as dermatan-binding protein PA-5541, was previously identified as being immunoreactive [26] and shares many properties with the above-mentioned protein PPA2127 (PA-25957). To unambiguously identify the stationary phase secretome of *P. acnes *future work is required to reduce the number of 'contaminating' (i.e. cytoplasmic) proteins; for instance, the choice of the culture medium might influence cell lysis. In addition, it is necessary for comparative reasons to determine the complete proteome of the cytoplasmic fraction.

## Conclusions

Despite the ubiquitous presence of *P. acnes*, our knowledge of this bacterium remains limited, in particular regarding the factors allowing its growth on human tissues. Many studies have shown that *P. acnes *has the ability to act as an opportunistic pathogen, with suggested etiological roles in a variety of inflammatory diseases. Due to its immune-stimulatory activity, it seems plausible that *P. acnes *causes inflammation within blocked sebaceous follicles or when it grows in tissue sites unaccustomed and/or hostile to this anaerobic bacterium. Hence, the ability of *P. acnes *to acquire and process growth substrates from its host, especially in the harsh environment of human skin, is dependent on the factors this bacterium secretes. The detection and identification of such factors are therefore important steps in further understanding *P. acnes *pathogenesis. Our study has highlighted the prevalence of secreted hydrolases likely to be involved in degrading human tissue components. Other identified proteins such as immunoreactive adhesins have a putative role in virulence. Secreted factors may also fulfill other functions such as defending against competing organisms and the evasion of the immune response. Functional characterization of these secreted factors is a necessary and logical next step, which requires the development of appropriate tools, e.g. a mutagenesis approach to create *P. acnes *knock-out mutants. Another challenge for the future lies in the elucidation of the molecular basis for observed differences in virulence between *P. acnes *isolates. The relationship between phylotypes (based on recA/tly sequences) and strain properties remains obscure; some properties, for instance the ability to trigger production of proinflammatory cytokines/chemokines in keratinocytes, seem to be phylotype-specific [21,22], whereas other properties, e.g. biofilm formation, are not [53]. Recent work has shown that an extended typing method based on serotyping in tandem with sequence comparison of three genes (trigger factor, p60, and mce) could distinguish invasive from non-invasive *P. acnes *isolates [54]; thus, this approach may be more appropriate for typing *P. acnes *isolates. In addition, our secretome analyses has revealed differences not only between but within phylotypes. A more extensive comparative analysis of *P. acnes *isolates incorporating robust phylotype identification will help to further our understanding of strain specificities.

## Methods

### Bacteria and growth conditions

The following *P. acnes *strains were used: 266 (type IA), P6 and KPA171202 (both type IB), 329 (type II), and 487 (type III). Strains 266, 329 and 487 were kindly provided by Oliver Knapp and Michel Popoff (Institut Pasteur). Strain KPA171202 was obtained from DSMZ (German German Collection of Microorganisms and Cell Cultures) and strain P6 was isolated from a cancerous prostate [55]. All *P. acnes *strains were cultured at 37°C on Brucella agar plates under anaerobic conditions for three days. Plate-grown bacteria were resuspended and washed in brain heart infusion (BHI) broth. Twenty ml BHI broth was inoculated with *P. acnes *(OD_600 _0.01) and grown for 12-72 h at 37°C and 160 rpm in an anaerobic jar. After 14-18 h, the cultures typically reached the mid-exponential growth phase with an OD_600 _of 0.5-0.6. Stationary phase was obtained after 72 h of growth.

### Precipitation of extracellular proteins

The exponential cultures were centrifuged for 15 min at 20,000 × *g *and 4°C, and the supernatant was filtered through a 0.22-μm-pore-size membrane filter to remove residual bacteria. Extracellular proteins were precipitated using a modified trichloroacetic acid (TCA) method as described previously [56]. In brief, the filtrate (100 ml) was mixed with 100% TCA to a final concentration of 10% and incubated overnight at 4°C. The mixture was centrifuged for 30 min (20,000 × *g *and 4°C) and the resulting pellet resuspended in 100 ml of acetone and dissolved using an ultrasonic water bath. The mixture was centrifuged as before, washed twice with acetone and the resulting pellet air dried.

### Two-dimensional gel electrophoresis

Protein samples were solubilized for 30 min at ambient temperature in 9 M urea-1% 3-[(3-cholamidopropyl)-dimethylammonio]-1-propanesulfonate (CHAPS)-70 mM dithiothreitol (DTT)-2% Servalyte 2-4 (Serva). Protein species were separated by a small-gel 2-DE system [57]. The samples containing 200 μg of protein were applied to the anodic side of the isoelectric focusing gel containing ampholytes in the pH range 2-11. The SDS-PAGE of the second dimension was performed using 15% acrylamide gels (7 cm × 8 cm). Protein spots were visualized by staining with Coomassie Brilliant Blue G-250 [58].

### MALDI-MS

Protein spots were identified by MALDI-MS after in-gel tryptic digestion of excised spots [59]. The peptide mixture was solubilized in 1 μl 33% acetonitrile/0.3% trifluoroacetic acid. For MALDI-MS measurement, 0.25 μl of the solubilized peptides were mixed with 0.75 ml a-cyano-4-hydroxycinnamic acid (CHCA) and spotted onto a MALDI plate. A 4700 Proteomics Analyzer (Applied Biosystems) with a mass range of 800-4000 Da was used for MS and at least 3 MS/MS spectra were measured per spot. Peptide mass fingerprinting (PMF) and MS/MS data were searched against the complete NCBI Database (Version 20090513). Proteins were identified using MASCOT 2.1 http://www.matrixscience.com allowing a peptide mass tolerance of 30 ppm and ± 0.3 Da for the fragment mass tolerance. A maximum of one missed cleavage, oxidation of methionine, N-terminal acetylation of the peptide, propionamide at cysteine residues and N-terminal pyroglutamic acid formation were considered in these searches. The identification criteria were: minimum 30% sequence coverage; or minimum 15% sequence coverage and one MS/MS confirmation; or sequence coverage below 15% and at least two MS/MS confirmations.

### DNA isolation, PCR and sequencing

DNA from *P. acnes *was isolated using the MasterPure™ Gram Positive DNA Purification Kit (Epicentre). Typing of *P. acnes *strains by *recA/tly *sequencing was performed as described previously [23]. For the analysis of the repetitive elements of PPA1880, PPA2127, and PPA2141 the PCR primers listed below were used to amplify 400-500 bps of the corresponding genomic region in strains P6, KPA and 266. PCR reactions were carried out using the Platinum Pfx DNA polymerase (Invitrogen), which has a proofreading 3'-5' exonuclease activity. PCR products were subsequently sequenced using the same primers. Primers: PPA1880_N_for CACTGTACGGACAGGTCTGG, PPA1880_N_rev CCATCCATATCGCACTTGTC; PPA1880_C_for GGCCAGCGAGACCTCTGATT, PPA1880_C_rev GGATGGGCAACAATTCGATG; PPA2127_N_for ATTCTCTACACGGCATGAGC, PPA2127_N_rev ATCCAGCCTTAACCAACGCA; PPA2127_C_for CAAGACTGCTGAGCAGCTCG, PPA2127_C_rev GCCGATGGTGATCAGAATCC; PPA2141_N_for CAACCTCGCTACGAAGTGGA, PPA2141_N_rev GGTCCTTGAGAACGGTATCG.

### Re-Annotation

All identified proteins were re-annotated, i.e. homology searches against sequence databases such as GenBank, and protein-domain/family databases, i.e. Pfam and InterPro, were performed. Homologous proteins in other bacteria were only discussed if sequence similarity to *P. acnes *proteins exceeded 25% on the protein level, with an overlap of the query and subject sequence of at least 90%.

### Accession numbers

The sequences reported in this study were deposited in GenBank. Sequences of *recA/tly *for the typing of the five strains have accession numbers HM461111 to HM461117. Sequence data from PPA1880, PPA2141, and PPA2127 have accession numbers HM461118 to HM461123.

## List of abbreviations

BHI: brain heart infusion; CAMP: Christie-Atkins-Munch-Petersen; GAPDH: glyceraldehyde 3-phosphate dehydrogenase; GehA: glycerol-ester hydrolase A; MALDI-MS: matrix-assisted-laser-desorption/ionization mass spectrometry; SP: signal peptide; TAT: twin-arginine translocation; 2-DE: two-dimensional gel electrophoresis.

## Authors' contributions

CH: protein sample preparations and data analyses; TNM: PCR analyses; UZA, MS, PRJ: 2-DE/MALDI-MS experiments and data analyses; CH, PRJ, TFM: assisted in the design of the study, and critically read the manuscript; HB: conceived and designed the study, analyzed data and wrote the manuscript. All authors read and approved the final manuscript.

## Supplementary Material

Additional file 1 **Secreted proteins of different *P. acnes *strains**. Bacteria were grown in BHI medium to an OD (600 nm) of 0.6. Proteins in the culture supernatants were precipitated using 10% TCA and separated on 2D-PAGE gels. (A) Second and (B) third replicate of the experiment shown in Figure 1 Click here for file

Additional file 2 **MS-based identification of all protein spots originating from exponential phase culture supernatants of five *P. acnes *strains**. This table lists all MS-identified proteins that were separated by 2-DE (see Figure 1). Click here for file

Additional file 3 **Alternative consequence of guanine stretch alterations upstream of PPA1880**. The homopolymeric guanine stretch could be part of the N-terminus of PPA1880. The different lengths of the G tract would lead to the formation of truncated proteins in strains KPA and 266 due to the appearance of a premature stop codon in the respective reading frame. Only in strain P6 a full protein would be synthesized. Click here for file

Additional file 4 **Adherence/agglutination of *P. acnes *strains grown to stationary phase**. 2 ml BHI medium per well was inoculated with the indicated five *P. acnes *strains (OD_600 nm _0.01) and grown to stationary phase (72 h) under anaerobic conditions (37°C, 110 rpm). Strain 266 agglutinated stronger than the other strains. Shown are two independent experiments. Click here for file

Additional file 5 **MS-based identification of all protein spots originating from the stationary phase culture supernatant of *P. acnes *strain 266**. This table lists all MS-identified proteins that were separated by 2-DE (see Figure 4). Click here for file
