# Supplementary figures and images for: Proteomic identification of secreted proteins of Propionibacterium acnes
Source: BMC Microbiol. 2010 Aug 27;10:230. doi: 10.1186/1471-2180-10-230 (PMC3224659; doi:10.1186/1471-2180-10-230)

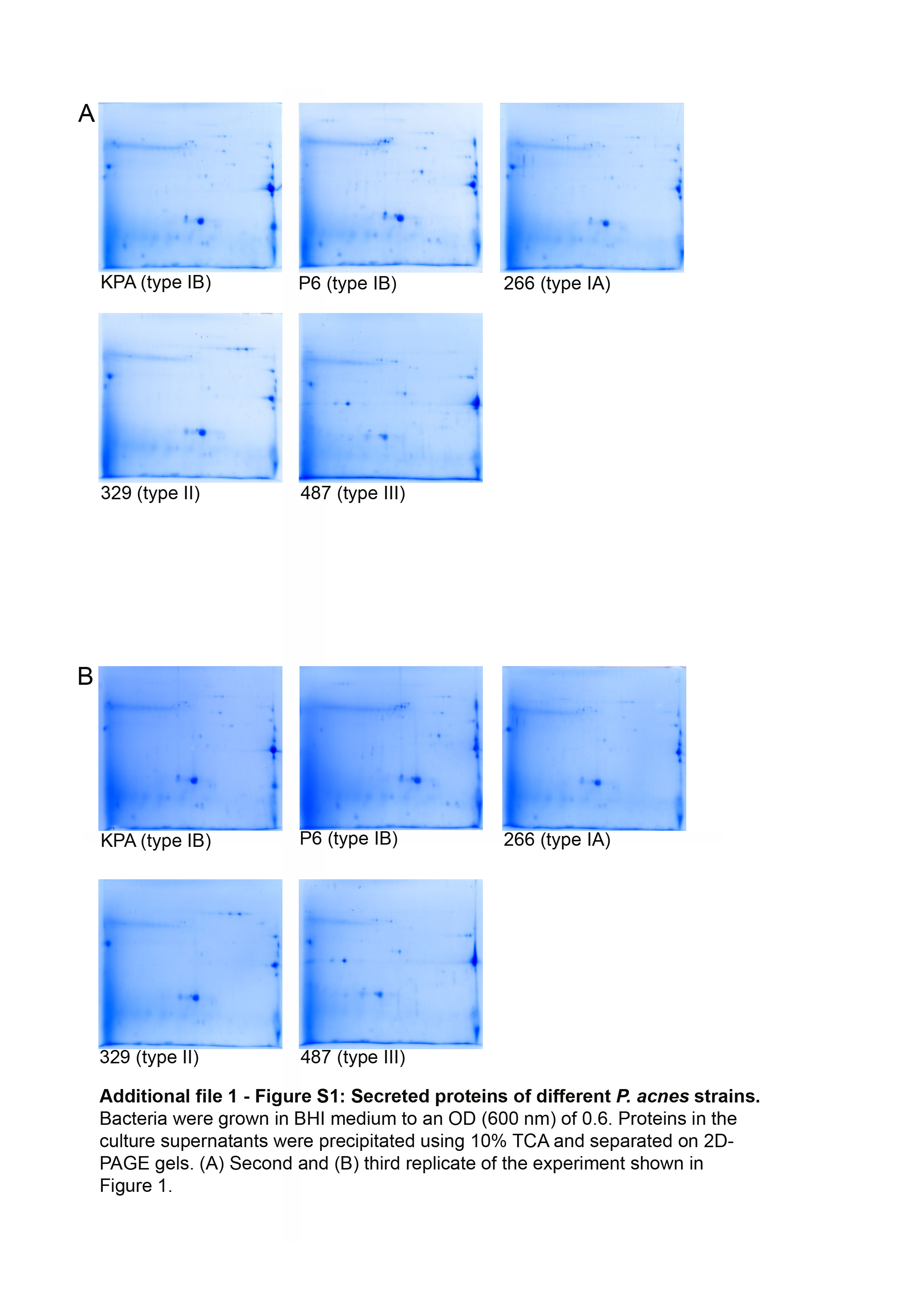

Supplement: Additional file 1 — Secreted proteins of different P. acnes strains. Bacteria were grown in BHI medium to an OD (600 nm) of 0.6. Proteins in the culture supernatants were precipitated using 10% TCA and separated on 2D-PAGE gels. (A) Second and (B) third replicate of the experiment shown in Figure 1 [file 1471-2180-10-230-S1.JPEG]
