# Supplementary material for: Proteomic identification of secreted proteins of Propionibacterium acnes
Source: BMC Microbiol. 2010 Aug 27;10:230. doi: 10.1186/1471-2180-10-230 (PMC3224659; doi:10.1186/1471-2180-10-230)
Supplement: Additional file 3 — Alternative consequence of guanine stretch alterations upstream of PPA1880. The homopolymeric guanine stretch could be part of the N-terminus of PPA1880. The different lengths of the G tract would lead to the formation of truncated proteins in strains KPA and 266 due to the appearance of a premature stop codon in the respective reading frame. Only in strain P6 a full protein would be synthesized. [file 1471-2180-10-230-S3.PDF]

Additional file 3 - Figure S2: Alternative consequence of guanine stretch alterations upstream of PPA1880.

The homopolymeric guanine stretch could be part of the N-terminus of PPA1880. The different lengths of the G tract would lead to the formation of truncated proteins in strains KPA and 266 due to the appearance of a premature stop codon in the respective reading frame. Only in strain P6 a full protein would be synthesized.

```
                                         V   G   H   L   C   S   A   S   Y   F
KPA   TCAGGTTTGTTTGAGGGGGTTGGGGTTTTGGG GTG GGG CAT TTG TGT TCT GCT AGC TAT TTT
                                         V   G   H   L   C   S   A   T   Y   F
266   TCAGGTTTGTTTGAGGGGGTTGGGGTTTTGGG GTG GGG CAT TTG TGT TCT GCT ACC TAT TTT
                                         V   G   H   L   C   S   A   S   Y   F
P6    TCAGGTTTGTTTGAGGGGGTTGGGGTTTTGGG GTG GGG CAT TTG TGT TCT GCT AGC TAT TTT

       M   N   D   V   D   K   I   A   G   G   G   G   Q   Y   F   *
KPA   ATG AAT GAT GTT GAC AAA ATC GCG GGG GGG GGG GGG CAG TAT TTC TAG CGC AGT TG
       M   N   D   V   D   K   I   G   G   G   G   G   G   Q   Y   F   *
266   ATG AAT GAT GTT GAC AAA ATC GGC GGG GGG GGG GGG GGG CAG TAT TTC TAG CGC AGT TG
       M   N   D   V   D   K   I   A   G   G   G   A   V   F   L   A   Q   L
P6    ATG AAT GAT GTT GAC AAA ATC GCG GGG GGG GGG GCA GTA TTT CTA GCG CAG TTG


KPA CCC GAT CCG TGG CTG TCA GAA GAA TGT CTG CGG CGC TGT ATG GCG CTT ACT GAA AGG

266 CCC GAT CCG TGG CTG TCA GAA GAA TGT CTG CGG CGC TGT ATG GCG CTT ACT GAA AGG
     P   D   P   W   L   S   E   E   C   L   R   R   C   M   A   L   T   E   R
P6  CCC GAT CCG TGG CTG TCA GAA GAA TGT CTG CGG CGC TGT ATG GCG CTT ACT GAA AGG
```
