# Supplementary material for: Proteomic identification of secreted proteins of Propionibacterium acnes
Source: BMC Microbiol. 2010 Aug 27;10:230. doi: 10.1186/1471-2180-10-230 (PMC3224659; doi:10.1186/1471-2180-10-230)

Additional file 4 - Figure S3: Adherence/agglutination of *P. acnes* strains grown to stationary phase. 2 ml BHI medium per well was inoculated with the indicated five *P. acnes* strains (OD_{600nm} 0.01) and grown to stationary phase (72 h) under anaerobic conditions (37 °C, 110 rpm). Strain 266 agglutinated stronger than the other strains. Shown are two independent experiments.

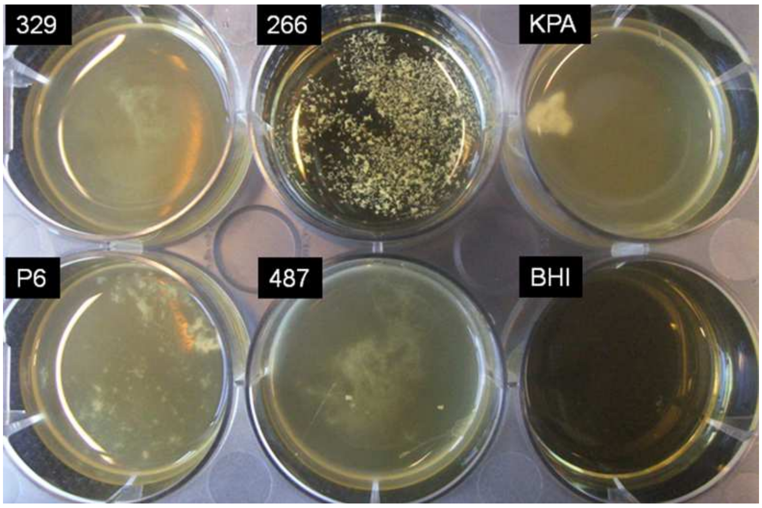

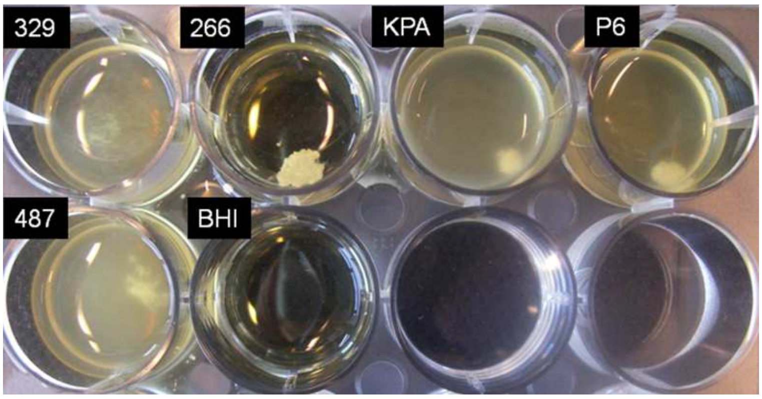

Supplement: Additional file 4 — Adherence/agglutination of P. acnes strains grown to stationary phase. 2 ml BHI medium per well was inoculated with the indicated five P. acnes strains (OD600 nm 0.01) and grown to stationary phase (72 h) under anaerobic conditions (37°C, 110 rpm). Strain 266 agglutinated stronger than the other strains. Shown are two independent experiments. [file 1471-2180-10-230-S4.PDF]
